# New York State Veterinary Medical Society

**Published:** 1890-09

**Authors:** W. P. Hinkley

**Affiliations:** Secretary


*New York State Veterinary Medical Society.*—The semi-annual meeting of the New York State Veterinary Medical Society was held at the Vanderbilt House, Syracuse, N. Y., July 15th and 16th, 1890. A large number of qualified veterinary surgeons from all parts of the Empire State were in attendance, showing that the society, although new in its organization, is one of the most solid and enterprising of the veterinary societies yet formed in our United States. President Morris called the meeting to order by a few well-chosen words, outlining the scope of the veterinary profession, its abuses in the past and its aims and wants in the future, also defining the class of men who have taken it up as a profession.

The roll was then called, showing nearly all of the organizers present. A recess was then taken for the censors to investigate the qualifications of applicants for membership. After the investigation by the censors, the following gentlemen were accepted and became members of the society:

John A. Bell, W. L. Baker, E. E. Bowen, James Carnrite, Charles Cowie, A. Drinkwater, W. G. Dodds, O. B. French, G. P. Geffery, W. G. Hollingworth, Wilson Huff, N. P. Hinkley, B. Howes, A. L. Hunter, J. J. Hill, E. D. Hayden, M. J. Henderson, E. B. Ingalls, H. C. Klicker, Prof. James Law, D. Leary, W. E. Langford, Asa N. McQueen, G. H. Moulter, C. D. Morris, M. M. Poucher, R. E. Rowell, F. A. Rich, G. H Roberts, B. K. Seltzer, William Somerville, Jr., Robert Somerville, Harry Sutterby, J. K. Sutterby, Frank Sutterby, W. S. Stevenson, G. H. Summerfeldt, John Wende, James Whytock, F. E. Williams, J. D. Whyte.

Letters of regret and telegrams of congratulation were read by the secretary from a number of prominent veterinary surgeons who were unable to be present at the meeting—one, in particular, from Prof. Liautard, regretting his inability to be present, on account of poor health, but stating his pleasure in seeing the organization of a veterinary society for our better protection and the advancement of the veterinary science, and sending us his sincere good wishes in our enterprise. The president then asked Prof. Law, of Ithaca, to make a few remarks. Prof. Law then spoke briefly on veterinary science and the want of better veterinary legislation, and of the need of better sanitary, police and scientific inspection of our meat, milk and other food products. He spoke of the extension of the disease actinomycosis, showing the necessity of better and more thorough inspection of our meats and stock, and the demand for more rigid laws by the government to stamp it out.

Prof. Law also thought it would be a good idea to have a veterinary examining board, similar to the Royal College of Veterinary Surgeons of England. Several of the veterinary surgeons in the room expressed their opinion and stated the urgent needs of veterinary surgeons on all our local health boards, and as inspectors of live stock, dressed meats, milk, and of all private and public dairies. They showed that a large percentage of our contagious diseases which our medical men have to cope with, are caused and produced by infected meats, impure milk, and unhealthy stables. The reports of standing committees were then received and accepted. Dr. Morris then reported the action taken at the meeting of our last Legislature. When the matter of better legisation came up before the society, every member in the room showed his individual interest by pledging himself by all honorable means to try and gain the assistance of all his friends to aid in petitioning the coming Legislature to grant us better laws and better protection for the qualified veterinary surgeon.

By-laws and a code of ethics were unanimously agreed upon by all members present, and adopted. The balance of the evening session was taken up by a number of the members communicating and describing important and interesting medical and surgical cases that had come under their personal observation in their recent practicc. The discussions caused by such communications were not only interesting, but were highly instructive to all members present, showing that the veterinary profession of to-day is composed of thoroughly educated and scientific men, who spend their time investigating and treating the numerous diseases and ailments of our live stock in a more scientific manner, and with better results, than has ever been known in past generations.

The question of papers for the next meeting, in January, 1891, was then discussed, and the following gentlemen were appointed to prepare them: Prof. James Law, Dr. John A. Bell, Dr. A. L. Hunter, Dr. H. Sutterby, Dr. A. McQueen.

The censors then met and filled out certificates and delivered them to members who were present. The meeting continued in active work until late in the afternoon of July 16th, when an adjournment was made to the second week of January, 1891, subject to the call of the secretary.

And thus closed two days of hard work of one of the most successful, enthusiastic and interesting meetings of veterinary surgeons ever held in the State of New York.

W. P. HINKLEY, *Secretary.*

